# Physiological Correspondence Between Different Indexes of High-Intensity Endurance Exercise in Young Male Runners

**DOI:** 10.3390/sports13060167

**Published:** 2025-05-29

**Authors:** Danilo A. Massini, Renato A. C. Caritá, Tiago A. F. Almeida, Anderson G. Macedo, Víctor Hernández-Beltrán, José M. Gamonales, Mário C. Espada, Dalton M. Pessôa Filho

**Affiliations:** 1 Department of Physical Education, School of Sciences, São Paulo State University (UNESP), Bauru 17033-360, Brazil; dmassini@hotmail.com (D.A.M.); tiagofalmeida.w@gmail.com (T.A.F.A.); andersongmacedo@yahoo.com.br (A.G.M.); dalton.pessoa-filho@unesp.br (D.M.P.F.); 2 Postgraduate Program in Human Development and Technology, São Paulo State University (UNESP), Rio Claro 13506-900, Brazil; 3 Faculty of Americana-(FAM), Americana 13477-360, Brazil; renatocarita@fam.edu.br; 4 Centre for Research in Economics and Comparative Development (CIEDEC), Lusíada University of Lisbon, 1349-001 Lisbon, Portugal; 5 Postgraduate Program in Rehabilitation Sciences, Institute of Motor Sciences, Federal University of Alfenas, Alfenas 37133-840, Brazil; 6 Training Optimization and Sports Performance Research Group (GOERD), Faculty of Sport Science, University of Extremadura, 10005 Cáceres, Spain; vhernandpw@alumnos.unex.es (V.H.-B.); martingamonales@unex.es (J.M.G.); 7 Faculty of Education and Psychology, University of Extremadura, 06006 Badajoz, Spain; 8 Instituto Politécnico de Setúbal, Escola Superior de Educação, 2914-504 Setúbal, Portugal; 9 Life Quality Research Centre (CIEQV-Leiria), Complexo Andaluz, Apartado, 2040-413 Rio Maior, Portugal; 10 Centre for the Study of Human Performance (CIPER), Faculdade de Motricidade Humana, Universidade de Lisboa, Cruz Quebrada-Dafundo, 1499-002 Lisboa, Portugal; 11 Comprehensive Health Research Centre (CHRC), Universidade de Évora, 7004-516 Évora, Portugal; 12 SPRINT Sport Physical Activity and Health Research & Innovation Center, Centro de Investigação e Inovação em Desporto Atividade Física e Saúde, 2001-904 Santarém, Portugal

**Keywords:** critical speed, respiratory compensation point, running, youth

## Abstract

Critical speed (CS), the respiratory compensation point (RCP), and the midpoint between gas exchange threshold and maxial oxygen uptake (VO_2max_) (i.e., 50%Δ) have been considered indexes able to demarcate the boundary between the heavy and severe exercise domains. However, the agreement between these indexes—and therefore the validity of using them reciprocally—remains to be reported in running. The current study analyzed the agreement between RCP, 50%Δ, and CS. Twelve young runners performed an incremental test to assess VO_2max_, RCP, and 50%Δ, with CS estimated by the linear model of time-limited trials at 90, 95, and 110% of the speed corresponding to VO_2_max. One-way ANOVA showed no differences when comparing VO_2_ and running speed at CS vs. 50%Δ vs. RCP (47.5 ± 4.4 vs. 46.6 ± 4.4 vs. 47.8 ± 4.5 mLO_2_∙kg^−1^∙min^−1^; and 13.9 ± 1.3 vs. 13.7 ± 1.3 vs. 14.0 ± 1.4 km∙h^−1^; *p* > 0.05 for all comparisons). The bias for 50%Δ vs. CS was −0.82 ± 1.55 mLO_2_∙kg^−1^∙min^−1^ and −0.23 ± 0.55 km∙h^−1^, and for RCP vs. CS, it was 0.36 ± 1.21 mLO_2_∙kg^−1^∙min^−1^ and 0.05 ± 0.46 km∙h^−1^. Therefore, the agreement between RCP, 50%Δ, and CS in estimating VO_2_ responses and running speed did not preclude their reciprocal similarity in exercise intensity, although the observed individual variability in physiological variables is a constraint on considering these indexes interchangeable.

## 1. Introduction

To define the zones for exercise training, the profiles of physiological variables like oxygen uptake (VO_2_) and blood lactate concentration ([La^−^]) during running or cycling have been used to account for the interplay of energy systems (aerobic vs. anaerobic activation) and acid–base balance control (metabolites (H+, Pi) clearance ability) [1,2]. Basically, the transition from an evenly steady state to an uncontrolled rise toward maximal values of VO_2_ and [La^−^], passing through an intermediary state of uneven but still controlled responses, characterizes the range of exercise intensities across the moderate (i.e., highly tolerable), heavy (fairly tolerable), and severe (poorly tolerable) domains [1,2,3]. To demarcate these domains, the responses of VO_2_, [La^−^], pulmonary ventilation (V_E_), and gas exchange variables (like carbon dioxide production (VCO_2_), ventilatory equivalents for O_2_ and CO_2_, and pulmonary end-tidal O_2_ and CO_2_) have been analyzed during incremental or constant-load exercises and applied to parametrize the transitions between physiological profiles in each exercise domain [4,5].

In this context, the onset of additional VCO_2_ release through the buffering process of H+ (due to the increased demand on the glycolytic pathway) drives [La^−^], V_E_, and VCO_2_ to rise more sharply, marking the transition from the moderate to heavy domain. This transition has been indexed by the lactate threshold (LT), the gas exchange threshold (GET), and the first ventilatory threshold (VT_1_) [4,5]. The heavy domain marks the upper limit of exercise at which blood lactate concentration ([La^−^]) and oxygen uptake (VO_2_) responses [2,3,4] remain high but stable, i.e., an isocapnic buffering region [5]. In this region, an exercise intensity can also be observed eliciting the maximal balance between the rate of metabolite efflux from the muscle and the rate of metabolite clearance from the blood (known as the maximal lactate steady state, MLSS) [2]. Above the heavy domain, the responses of [La^−^] and VO_2_ are projected to their respective maximal values [6,7]; thus, the transition from the heavy to severe domain is characterized by respiratory compensation for metabolic acidosis (i.e., V_E_ rises exponentially (hyperventilation) to reduce the excess of CO_2_ in the blood), which has been indexed by the lactate turn point (LTP), respiratory compensation point (RCP), and second ventilatory threshold (VT_2_) [4,5].

In running, critical speed (CS) has been recognized and well supported as the exercise intensity demarcating the upper limit of the heavy domain [2,6,7,8]. Thus, below CS, there is a range of exercise intensities eliciting physiological [1,2,8] and metabolic [2,3,9] responses without inducing sufficient accumulation of metabolites to disturb the acid–base balance [9,10]. However, the determination of CS yields values subjected to protocol influence (i.e., time limit (t_Lim_) between 2 and 15 min) [1,2,3] and mathematical adjustments (linear and non-linear equations) [2,11] and is often expensive and physically demanding for athletes [12].

In addition, other indexes able to parametrize the zone of heavy but still sustainable exercise, mainly those assessed during incremental exercise tests, have been compared to CS regarding their observed similarities [6,13]. For example, it has been proposed that the exercise intensity at half the difference (i.e., Δ 50%) between maximal oxygen consumption (VO_2max_) and GET would also delimit the upper boundary of exercise intensities with physiological responses still characterizing the heavy domain [14]. Despite all normalization efforts, information from incremental exercise tests is dependent on the specificity of training status and movement pattern [15,16,17,18]. Lansley et al. [19] reported more consistent physiological responses during exercise when the level of intensity was prescribed at a given %Δ rather than at %VO_2max_. Moreover, another study reported that the transition from the heavy to severe domain in running can be observed in a wide range between 40 and 60%Δ [20]. However, typical physiological responses of the heavy domain were reported during cycling at 50%Δ [14], whereas responses at 60, 70, and 80%Δ were typical of the physiological profile in the severe domain [21]. Therefore, there is enough theoretical support for the assumption that 50%Δ might be a reliable index of the transition between sustainable and exhaustive exercises [14].

As already mentioned, another index from the incremental exercise test able to characterize the heavy sustainable exercise intensity is RCP [22,23], which has shown similarity to CS [3,11]. The physiological significance of RCP corresponds to an exercise intensity beyond which the mechanisms for controlling the acid–base balance lose the capacity to buffer hydrogen anion production due to the increased demand for ATP resynthesis from anaerobic glycolytic metabolism [4,5,10,11,13]. Thus, the concept of RCP is best aligned with the assumption that it represents an exercise intensity around which the physiological response can transition from the heavy to severe domain [24,25]. Therefore, if CS is an index from a time-limited model able to evidence agreement with threshold indexes of incremental exercise, then CS can be a reliable and feasible index to support high-intensity training planning for young runners. It is notable that the insufficient number of investigations to support the agreement or disagreement between CS and indexes from incremental exercise, particularly RCP and 50%Δ, contrasts with the substantial evidence supporting their roles in demarcating exercise zones characterized by submaximal and sustainable physiological responses. In addition, previous studies did not refute that both RCP and CS are indexes eliciting comparable load/speed intensities or physiological responses [11,26], although the interchangeability in physiological responses is still being questioned [5,18].

Thus, the present study aimed to analyze metabolic (VO_2_) and speed (km·h^−1^) agreement at 50%Δ and RCP with CS during treadmill running in trained young runners. The hypothesis was that these indexes would show statistical similarities in terms of oxidative demand. However, when comparing running speeds, differences were expected to reduce compatibility due to the influence of individual anthropometric characteristics on stride length and frequency and their association with running economy [17].

## 2. Materials and Methods

### 2.1. Participants

Twelve young male runners (15.7 ± 1.8 years; 1.7 ± 0.1 m; and 57.1 ± 11.7 kg), regularly involved in a running training program for at least two years, participated in this study. Assessments were conducted during the third microcycle of the base training period. The participants were selected after an initial screening of 20 young athletes to exclude those who were recently injured or sick, those experiencing pain or discomfort during the training, and those who were not endurance-trained runners. All participants obtained permission from their guardians and signed an informed consent form acknowledging the procedures. All research procedures were conducted following the Declaration of Helsinki and the ethical standards in sport and exercise science research [27], and were previously approved by the local University Ethics Committee (CAEE: 36936714.6.0000.5398).

### 2.2. Experimental Design

**Figure 1:**
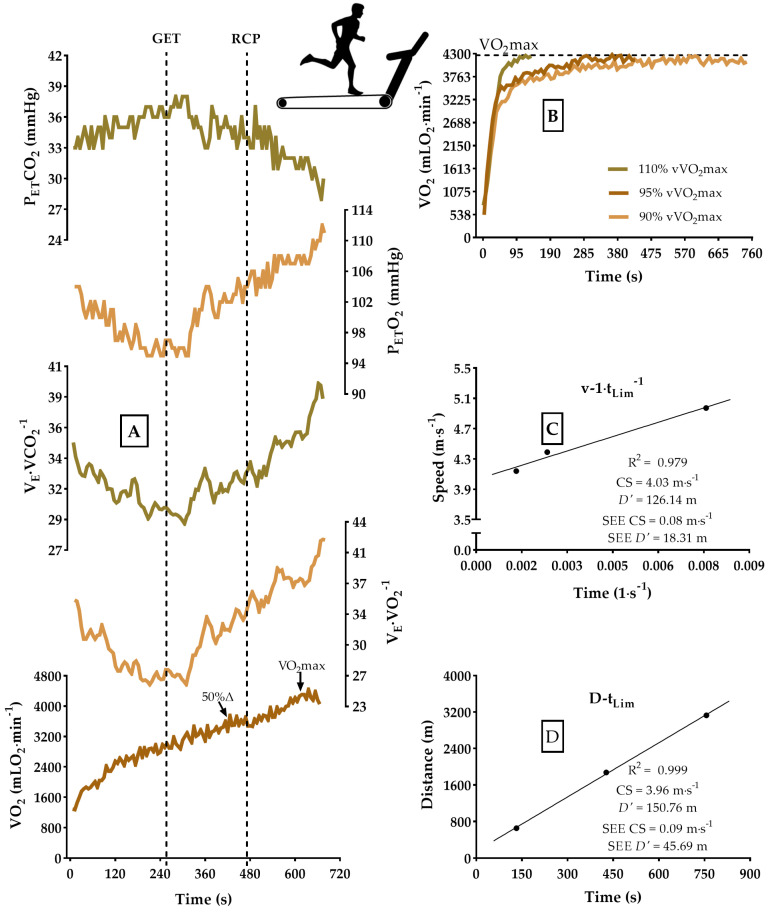
An example of estimating a participant’s physiological and speed variables in the study. Panel (**A**) depicts the maximal incremental test for determining GET, RCP, 50%∆, and VO_2max_. Panel (**B**) illustrates the VO_2_ response during the predictive trials for CS. Panels (**C**,**D**) show the determination of CS using the v-tLim^−1^ and d-t_Lim_ models, respectively.

Figure 1 illustrates the experimental design. Participants underwent four testing sessions: (1) a progressive ramp test to determine GET, RCP, 50%Δ, and VO_2_max, along with their respective intensities (vGET, vRCP, v50%Δ, and vVO_2_max, respectively); and (2) three constant-velocity exercise bouts to voluntary exhaustion at 90%, 95%, and 110% of vVO_2_max, which were performed for CS prediction. A 24 h interval was maintained between each exercise bout [8]. All tests were conducted on a motorized treadmill (HP/Cosmos Pulsar, Nussdorf-Traunstein, Germany) with a fixed 1.0% incline [26] in an indoor environment with controlled temperature (21–23 °C) and air humidity (50–60%). Participants were instructed to avoid exhaustive training, refrain from drinking alcoholic and caffeinated beverages the day before the assessment, and arrive in a fed and hydrated state [7,8].

### 2.3. Maximal Incremental Test

During the maximal incremental test (Figure 1, Panel A), the speed progressed by 1.0 km∙h^−1^∙min^−1^, starting from 7.0 km∙h^−1^ [28] until voluntary exhaustion. VO_2_ was breath-by-breath sampled throughout the test (QuarkPFTergo, Cosmed, Rome, Italy). The O_2_ and CO_2_ concentration analysis system was calibrated before each test using ambient air and a gas with known O_2_ and CO_2_ concentrations, and the turbine analysis was calibrated using a three-liter syringe, according to the manufacturer’s recommendations. VO_2_ values were smoothed by a three-second filter and averaged every six seconds. [La^−^] was analyzed at rest and in the first minute after test completion, and its analysis was performed using the enzymatic method (YSL 2500STAT, Yellow Spring, CO, USA) from 25 μL of arterial blood diluted in 50 μL of 1% NaF solution [11].

VO_2_max was considered the highest value smoothed by a 30 s moving average. VO_2_max was determined as the lowest speed in the incremental test that elicited the maximum VO_2_ elevation [17]. GET was determined following Whipp’s [4] recommendations based on the responses of V_E_∙VCO_2_^−1^, V_E_∙VO_2_^−1^, P_ET_CO_2_, and P_ET_O_2_. It involved observing an increase in the responses of VE∙VO_2_^−1^ and P_ET_O_2_ without a change in the response of V_E_∙VCO_2_^−1^ and P_ET_CO_2_. Identification of the metabolic fatigue threshold at 50%Δ was performed using VO_2_ at GET and VO_2max_ (50%Δ = GET + [(VO_2max_ − GET) × 0.5]) [14,16]. The corresponding speed was determined by trend fitting between running speed (km∙h^−1^) and its VO_2_ during the incremental test. GET, RCP, 50%Δ, and VO_2max_ determinations were conducted independently by three experienced researchers [29].

### 2.4. Critical Speed (CS) Determination

The predictive trials for CS (90%, 95%, and 110% of vVO_2max_) (Figure 1, Panel B) were randomly performed with a minimum interval of 24 h [8]. The value of t_Lim_ was recorded in seconds and associated with the prediction speed using the speed vs. time-limited (v-t_Lim_^−1^) model (Figure 1, Panel C) and the distance vs. time-limited (d-t_Lim_) model (Figure 1, Panel D) (Equations (1) and (2)) [12]. The selection of the CS value for each participant was based on the smallest standard error of the estimate (SEE) as the criterion [30].

(a) Speed vs. inverse of time to exhaustion (v-1·t_Lim_^−1^)

(1) $$v=D^{\prime }\times \frac{1}{\left(t\right)}+CS$$

(b) Total distance vs. time (D-t_Lim_)

(2) $$d=D^{\prime }\times t+CS$$

where *v* = running speed; *D*′ = amount of work from bioenergetic reserves; *t* = the time limit (t_Lim_) of predictive trials; and *CS* = critical speed. The VO_2_ corresponding to CS was determined using the trend relationship between VO_2_ and running speed during the incremental test [7,30].

### 2.5. Statistical Analysis

The sample size was previously estimated using G*Power, considering a security level of 95% (Z_1_-α/2 = 1.960) and power of 85% (Z_1_-β = 1.036), as well as a high correlation level of 0.80 [31]. The estimated size was 10 participants, which was increased by 20% (N = 12) to avoid statistical underpower with participants withdrawing.

Data were expressed as mean ± SD with a 95% confidence interval (CI_95%_). Outliers and normality were checked using a 1.5∙IQR range (interquartile range = Q3–Q1) and the Shapiro–Wilk test. Homogeneity, variance, and differences between 50%Δ, RCP, and CS (VO_2_ and km∙h^−1^) analysis were examined using the Levene test and one-way ANOVA, supplemented by Fisher’s LSD test.

The normality, independence, and homoscedasticity of the residuals for the regression analyses between 50%Δ, RCP, and CS were assessed using the Shapiro–Wilk, Durbin–Watson, and Breusch–Pagan tests, respectively. Influential residuals or leverage points were identified through Cook’s distance. For agreement analysis, the difference between the means of variables was checked using the two-tailed Student’s *t*-test for a single sample, and the proportional bias of differences was assessed through linear regression analysis between the mean (independent variable) and the difference (dependent variable) of the measurement. An analysis of dispersion was performed using the sample-adjusted coefficient of determination (R^2^adj.) and SEE, whilst agreement was analyzed with Bland–Altman plots [32] and the standard error of the mean (SEM = SD ÷ √n).

The effect size for ANOVA and R^2^adj. was calculated using eta squared (η^2^) considering threshold values as follows: <0.04 [trivial], 0.04–0.24 [weak], 0.25–0.63 [medium], and ≥0.64 [strong] [33]. The correlations between the variables were assessed using the coefficient of correlation (r) derived from the coefficient of determination (R^2^), with which the sample power was determined (Equation (3)) [31].

(3) Z1−β=n−3 12Ln1+r1−r−Z1−α2

where *Z*_1−*β*_ provides the coefficient for determining the sample power by the bicaudal normal distribution of the value *r* [31]. The IBM SPSS Statistics software (version 27, 2021, IBM Co., Ltd., Armonk, NY, USA) was used to conduct statistical analyses. Statistical significance was determined at α < 0.05.

## 3. Results

**Figure 2:**
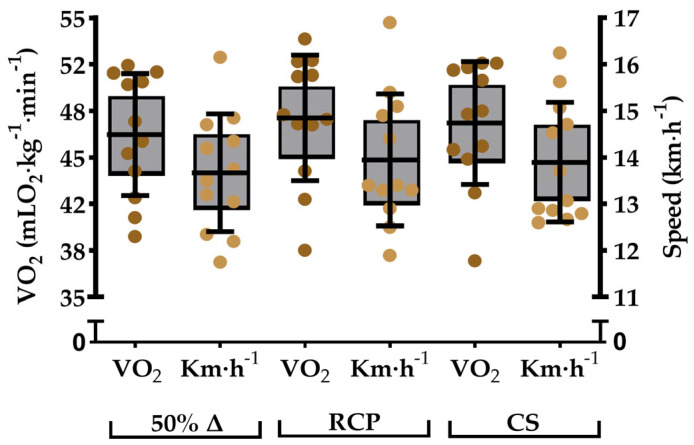
Individual values (circles), mean ± SD (line and whiskers), and 95% confidence interval (gray box) of VO_2_^−^ (brown) and running speed (light brown) associated with 50%∆, RCP, and CS.

The value of VO_2max_ reached 53.0 ± 5.1 (CI_95%_ = 49.8–56.2) mLO_2_∙kg^−1^∙min^−1^, and the corresponding speed reached 16.1 ± 1.7 (CI_95%_ = 15.0–17.2) km∙h^−1^. The lower limit of the heavy domain was indexed by GET, corresponding to 76.2 ± 4.2% VO_2_max (70.1 ± 6.2% vVO_2_max), and CS marked the upper limit at 89.6 ± 3.1% VO_2_max (86.5 ± 3.6% vVO_2_max). The VO_2_ responses during predictive trials for CS reached 53.0 ± 4.9 (110% vVO_2_max), 53.9 ± 4.4 (95% vVO_2_max), and 56.2 ± 7.2 mLO_2_∙kg^−1^∙min^−1^ (90% vVO_2max_), providing greater prediction rigor for the v^−1^∙t_Lim_^−1^ fit with R^2^ = 0.98 ± 0.03 (SEE = 2.89 ± 1.69% and CI_95%_ = 0.25–0.55 km∙h^−1^). Figure 2 presents the metabolic and speed values of the indexes used in the upper limit of the heavy domain. Another variable for sustainable exercise was RCP, which was situated at 90.2 ± 2.8% VO_2_max (86.8 ± 3.7% vVO_2_max). Both 50%Δ (88.0 ± 3.7% VO_2max_ and 85.1 ± 3.1% vVO_2max_) and RCP showed no significant differences when compared to CS, respectively, regarding VO_2_ (F_[2,35]_ = 0.227, η^2^ = 0.014 [trivial], *p* = 0.65 and *p* = 0.84, respectively) and running speed (F_[2,35]_ = 0.151, η^2^ = 0.009 [trivial], *p* = 0.68 and *p* = 0.92, respectively). Similarly, there was no difference in RCP compared to 50%Δ for VO_2_ (*p* = 0.51) and km∙h^−1^ (*p* = 0.61), confirming the hypothesis regarding the statistical similarity between them.

**Figure 3:**
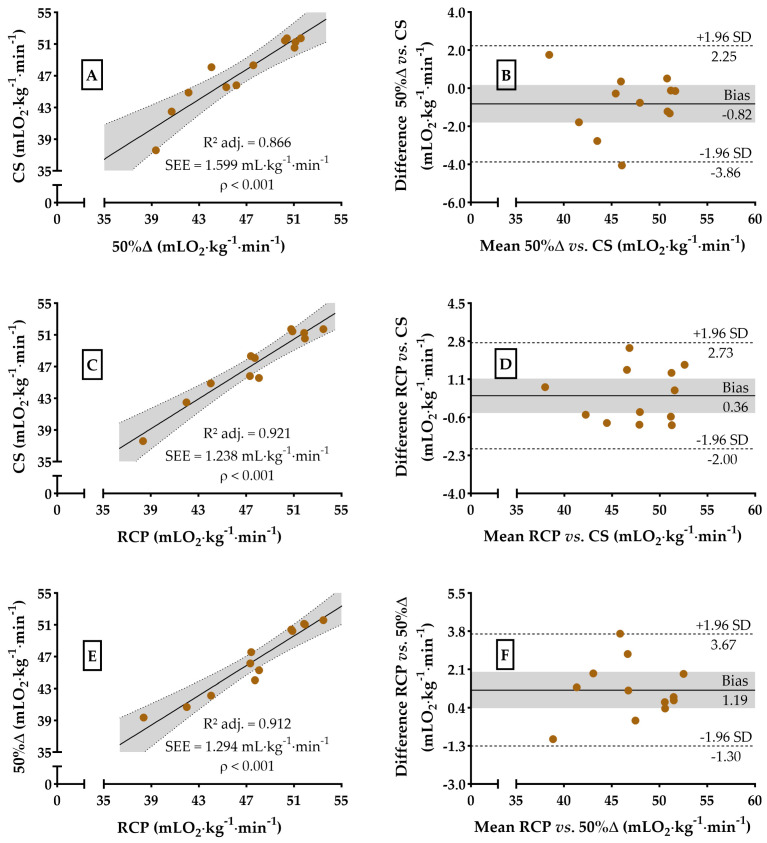
Dispersion and Bland–Altman plots for VO_2_ values at CS, RCP, and 50%∆. Dispersion between VO_2_ at 50%∆ vs. CS (99.9% power, Panel (**A**)), VO_2_ at RCP vs. CS (99.9% power, Panel (**C**)), and VO_2_ at RCP vs. 50%∆ (99.9% power, Panel (**E**)). Agreements analysis between VO_2_ at 50%∆ vs. CS (Panel (**B**)), RCP vs. CS (Panel (**D**)), and RCP vs. 50%∆ (Panel (**F**)). The gray area in all the panels represents the 95% confidence interval.

**Figure 4:**
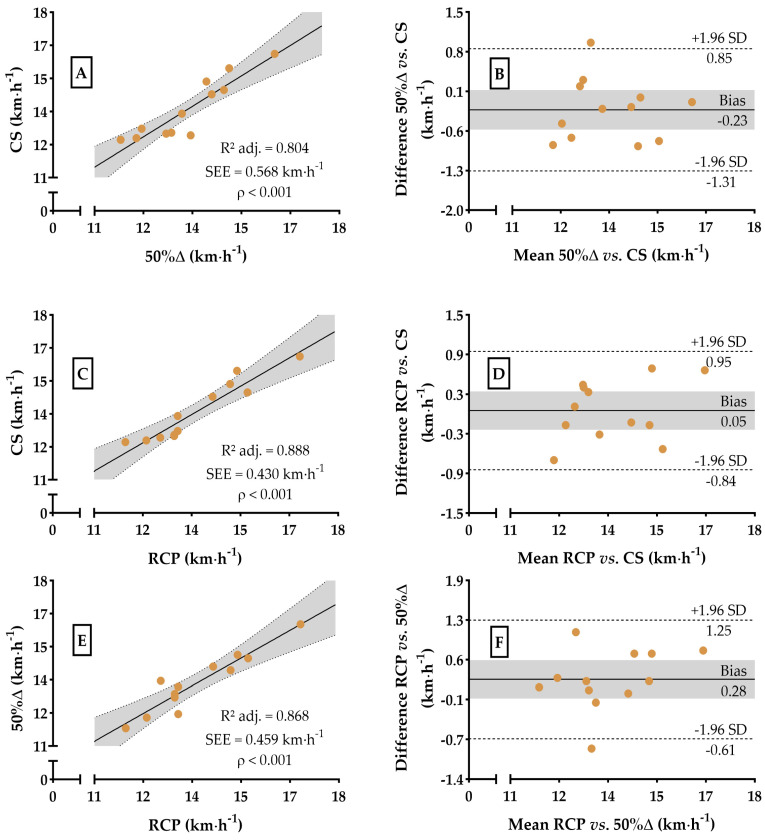
Dispersion and Bland–Altman plots for speed (km∙h^−1^) values at CS, RCP, and 50%∆. Dispersion for 50%∆ vs. CS (99.4% power, Panel (**A**)), RCP vs. CS (99.9% power, Panel (**C**)), and RCP vs. 50%∆ (99.9% power, Panel (**E**)). Agreements analysis between velocities at 50%∆ vs. CS (Panel (**B**)), RCP vs. CS (Panel (**D**)), and RCP vs. 50%∆ (Panel (**F**)). The gray area in all the panels represents the 95% confidence interval.

**Figure 5:**
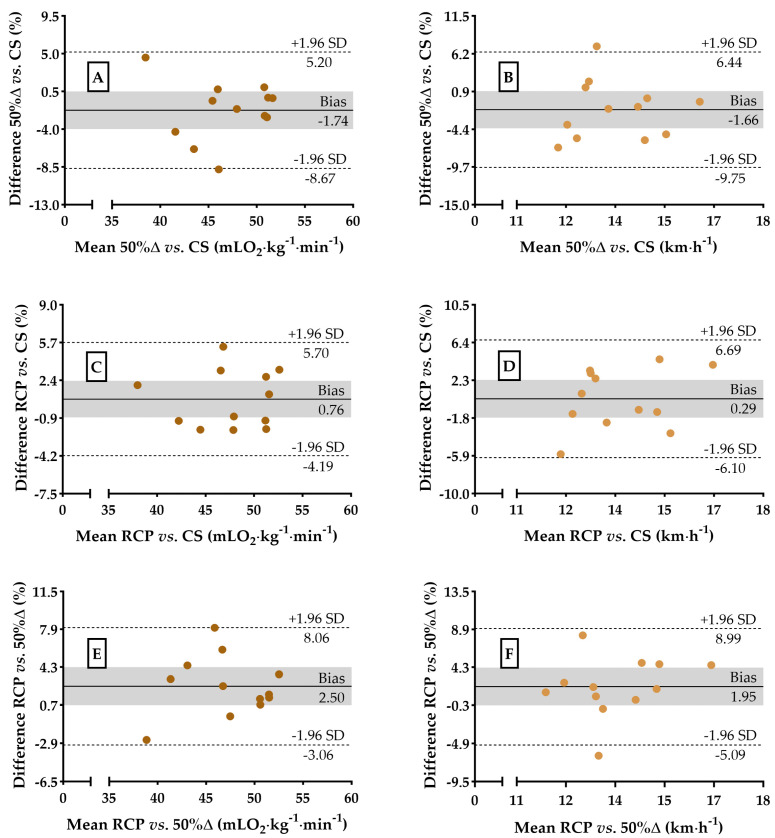
Bland–Altman plots showing the percentage difference between VO_2_ values and running speed (respectively) for 50%∆ vs. CS (Panel (**A**,**B**)), RCP vs. 50%∆ (Panel (**C**,**D**)), and RCP vs. 50%∆ (Panel (**E**,**F**)). The gray area in all the panels represents the 95% confidence interval.

The regression and agreement analyses between 50%Δ, RCP, and CS regarding VO_2_ and running speed are shown in Figure 3 and Figure 4, respectively. The agreement analyses in relative values of VO_2_ and km∙h^−1^ are shown in Figure 5. Both 50%Δ (F_[1,10]_ = 72.13, *p* < 0.01) and RCP (F_[1,10]_ = 128.6, *p* < 0.01) were able to account for the variance in VO_2_ at CS with a potential of 86.6% [strong] and 92.1% [strong], respectively (Figure 3A,C), and a bias (t_[11]_ = −1.830, *p* = 0.94 and F_[1,11]_ = 0.003, *p* = 0.96; t_[11]_ = 1.046, *p* = 0.32 and F_[1,11]_ = 0.065, *p* = 0.80) of −0.82 ± 1.55 and 0.36 ± 1.21 mLO_2_∙kg^−1^∙min^−1^ (SEM = 3.0 and 2.4 mLO_2_∙kg^−1^∙min^−1^ or 6.9 and 4.9%, respectively, Figure 3B,D) or −1.74 ± 3.54 and 0.76 ± 2.52%, respectively (t_[11]_ = −1.701, *p* = 0.12 and F_[1,11]_ = 0.004, *p* = 0.94; t_[11]_ = 1.041, *p* = 0.32 and F_[1,11]_ = 0.016, *p* = 0.90, Figure 5A,C).

On the other hand, RCP (F_[1,10]_ = 115. 3, *p* < 0.01) has the potential to predict VO_2_ at 50%Δ, explaining 92.1% of the variance [strong] (Figure 3E), but lacks statistical agreement (t_[11]_ = 3.234, *p* < 0.01 and F_[1,11]_ = 0.096, *p* = 0.76), with a bias of 1.19 ± 1.27 mLO_2_∙kg^−1^∙min^−1^ (SEM = 2.5 mLO_2_∙kg^−1^∙min^−1^ or 5.5%, Figure 3F) or 2.50 ± 2.84% (t_[11]_ = 3.049, *p* = 0.01 and F_[1,11]_ = 0.037, *p* = 0.85, Figure 5E). For running speed, a lack of statistical compatibilities was also observed, confirming our second hypothesis. Both 50%Δ (F_[1,10]_ = 47.16, *p* < 0.01) and RCP (F_[1,10]_ = 87.84, *p* < 0.01) were able to account for the variance in km∙h^−1^ at CS with a potential of 80.4% [strong] and 88.8% [strong], respectively (Figure 4A,C), and a bias (t_[11]_ = −1.431, *p* = 0.18 and F_[1,11]_ = 0.014, *p* = 0.91; t_[11]_ = 0.392, *p* = 0.70 and F_[1,11]_ = 0.957, *p* = 0.35) of −0.23 ± 0.55 and 0.05 ± 0.46 km∙h^−1^ (SEM = 1.0 and 0.9 km∙h^−1^ or 8.0 and 4.9%, respectively, Figure 4B,D) or −1.66 ± 4.13 and 0.29 ± 3.26%, respectively (t_[11]_ = −1.391, *p* = 0.19 and F_[1,11]_ = 0.006, *p* = 0.94; t_[11]_ = 0.310, *p* = 0.76 and F_[1,11]_ = 0.835, *p* = 0.38, Figure 5B,D).

Finally, RCP (F_[1,10]_ = 73.51, *p* < 0.01) explained 86.8% of the variance in running speed (km∙h^−1^) at 50%Δ [strong] (Figure 5E), with a bias (t_[11]_ = 1.955, *p* = 0.08 and F_[1,11]_ = 1.095, *p* = 0.32) of 0.28 ± 0.49 km∙h^−1^ (SEM = 1.0 km∙h^−1^ or 7.0%, Figure 4F) or 1.95 ± 3.54% (t_[11]_ = 1880, *p* = 0.09 and F_[1,11]_ = 0.541, *p* = 0.48, Figure 5F).

## 4. Discussion

This study analyzes the agreement between physiological indexes in running speed at different fatigue thresholds in young runners. By assessing 50%∆ and RCP during an incremental ramp test, no differences were significant when comparing these indexes in terms of metabolic rate and running speed, nor when comparing these responses from both indexes with those estimated for CS. In light of these findings, this study demonstrates that all three indexes might be applied to determine the transition between the heavy and severe exercise intensity domains for young runners, showing similarities with the age group and training status of the participants. However, the Bland–Altman analysis [32] showed wide and biased confidence intervals, indicating error in metabolic rate and speed between 50%∆ and CS, which is a finding consistent with previously reported variability in cycling [12]. Also, the speeds corresponding to 50%∆ and RCP were not in agreement (i.e., comparisons differed from zero significantly) and showed a considerably high estimation error (7.0%). Since the errors exceed 5%, the planning of training (e.g., targeting an athlete’s periodization) and/or the design of experimental protocols (e.g., targeting analysis of exercise intensity) may not provide a reliable estimate of exercise conditions [12,13]. Indeed, previous research has suggested that exercising at 5% above CP reduces tolerance and elicits muscular and blood metabolic imbalance [34]. Taking this into account, only the comparisons of VO_2_ and speed between RCP and CS might be considered closely reciprocal and likely to provide high confidence in determining different exercise domains around these thresholds.

Thus, the lack of statistical differences between the indexes (RCP vs. 50%Δ vs. CS) regarding the VO_2_ response should be interpreted with caution since the individual variability of the responses observed might preclude the assumption of similar metabolic responses for the entire sample of participants in each index, in line with previous studies reporting a lack of similarity in cycling [13,26,35] and running [6]. Individual variability in physiological thresholds across exercise domains has been reported to be influenced by slight differences in training experience, conditioning level, and running economy [10,35,36]. For example, altering the slope of the relationship between VO_2_ and speed to the right allows a previously required metabolic demand to be maintained at a higher running speed or enables a given running speed to be sustained for a longer duration [10]. Although this tendency is well reported for training in the moderate exercise domain, there is a large variance among individuals regarding the changes, as was observed by Philp et al. [36] after four weeks of training with moderately trained runners (VO_2max_ = 45 mLO_2_·min^−1^·kg^−1^), in which the increases in the lactate threshold (LT) (7–9%), maximal lactate steady state (5–8%), and VO_2max_ (6–10%) showed variability. Consequently, the relative allocation (%VO_2max_ and/or %vVO_2max_) of the physiological thresholds changes, and the boundaries of the heavy domain (lower and upper, e.g., LT, CS) move up accordingly [16]. In addition, further considering that the relative location of these indexes depends on individual adaptation to training [19,37], as well as on the sensitivity of each index to adjustments based on the specialized plan of training [16], the interchangeability between RCP, 50%Δ, and CS remains a critical concern, due to the particular contribution of energetics (aerobic vs. anaerobic) and tolerance to exercise [25,38], even when differences in exercise intensity among these indexes are slight [13,18]. However, the current study observed very close values at a given %VO_2max_ and %vVO_2max_ between RCP and CS, suggesting that exercise 1% below and/or above these indexes tends to fall in different domains, which indicates a closer overlap of boundaries.

Therefore, an aspect to consider is individual variability, which is intrinsic to the relative allocation of RCP, 50%Δ, and CS in terms of %VO_2max_ and/or %vVO_2max_. The current study observed a very small underestimation of CS by 50%Δ (~1.6%). When observing the current results in terms of Δ, two participants had CS located above 60%Δ, which would imply an underestimation of the upper limit of the heavy domain by 50%Δ. However, considering the study of Lansley et al. [19] recommending a safety margin (±10%Δ) to define the boundary between heavy and severe domains based on 50%Δ, the current mean difference between CS and 50%Δ is well aligned with this suggestion, supporting the use of 50%Δ to parametrize exercise domains. However, it is important to note the probability of observing different physiological responses (VO_2_ and heart rate), metabolic responses ([La^−^]), and exercise tolerance between individuals when predicting exercise intensity in a given domain [1,34]. An attempt to account for this variability was proposed by Souza et al. [12], whose study suggested an even larger “safe zone” of ±20%Δ (i.e., heavy domain <30%Δ and severe >70%Δ) to accommodate major sources of interindividual variability in physiological responses, such as (i) training status [35]; (ii) central and peripheral limitations to oxygen uptake during exercise, muscle fiber contractile properties, and metabolic profile [18]; and (iii) age and sex [35]. In spite of the possible large variability in physiological responses reported in these previous studies when exercising at different paces near the boundary between the heavy and severe domains, the current study emphasizes that the proportional similarity between CS and 50%Δ is strong (i.e., showing a rate above 80%) for speed or VO_2_ comparisons.

In addition, the comparison between RCP and CS showed the strongest similarities (~90%) when comparing the speed and estimated VO_2_ for these indexes. Nevertheless, the SEM can attain ~5%, which might result in allocating RCP within the severe intensity domain in different sport modalities, such as running, cycling [1,2,4,24], and swimming [23]. There are many studies comparing RCP with CS (or CO, which is the equivalent for power measurements) that have observed statistical equivalence, as shown in cycling (286 ± 28 W or 85.3 ± 5.6% VO_2_max vs. 278 ± 22 W or 85.4 ± 4.8% VO_2_max, respectively) [22], in running (RCP vs. CS: 11.5 ± 2.3 vs. 11.7 ± 2.3 km∙h^−1^, respectively) [6], and in oxidative demand while running (2.88 ± 0.80 vs. 2.83 ± 0.72 L∙min^−1^, respectively) [6]. Therefore, the current results reinforce the lack of difference between RCP and CS, in line with previous findings [22,23] for either absolute (VO_2_ and speed) or relative (%VO_2max_ and/or %vVO_2max_) measures. In practical terms, the closest similarities between RCP and CS suggest a reciprocal confidence for planning high-intensity exercise in the heavy and severe domains, with each index being suitable for control running intensity during training and conditioning adjustments over a planning period, despite the availability of a metabolic chart being mandatory for RCP assessment.

The current study showed some limitations regarding the measurements, experimental conditions, and population. The first concern is related to the assessment of CS, since the variability of the values is associated with the number of predictive trials [2] and mathematical adjustments (linear and non-linear) [2,30]. However, the current study ensured that the t_Lim_ of the predictive trials lay around the recommended range (2 to 15 min), and that VO_2_max was reached during the trials at 90%, 95%, and 110% VO_2max_ [2,6,9]. The fact that VO_2_ responses were not analyzed during exercise (e.g., VO_2_ on-kinetics) around each index (RCP, 50%Δ, and CS) limits the ability to assert metabolic equivalence between these indexes and prevents a definitive recommendation regarding their interchangeability for demarcating the transition from the heavy to severe exercise domain. In addition, the findings of the current study are restricted to the analyzed group regarding the age and training level of the runners; therefore, extrapolation of the results to a larger sample of young runners should be interpreted with caution. Moreover, we recommend future studies investigating the similarity between different training conditions and age groups of runners.

## 5. Conclusions

The results indicated that during maximal progressive exercise, certain indexes of speed and VO_2_ demand were highly correlated with CS, suggesting their ability to represent the transition from high-intensity, yet sustainable, exercise to poorly tolerated zones. Therefore, the indexes 50%Δ and RCP can be considered useful references for identifying the transition between heavy and severe exercise domains, due to their physiological (VO_2_) and speed (km∙h^−1^) similarities with CS. However, interindividual variabilities should be considered a source of estimation errors of these variables, and, thus, the interchangeability between these indexes must be interpreted with caution. This consideration is essential for planning training and experimental designs demanding a high level of specificity regarding the physiological responses in young runners, since large variability might allocate different subjects in distinct exercise zones. In addition, the current analysis also evidenced that CS might be an excellent index for characterizing the heavy domain in athletes of comparable age and training levels (or experience) to the present participants, considering that RCP and 50%Δ both showed a tendency to overestimate CS. Therefore, although the reliability of RCP, 50%Δ, and CS for planning training in the heavy and severe domains for young runners remains a questionable issue due to the lack of detailed physiological comparisons among these indexes, the current findings support a high degree of similarity in exercise intensity, at least from running speed comparisons, between RCP and CS.

## Data Availability

The data that support the findings of this study are available from the corresponding and last author (dalton.pessoa-filho@unesp.br) upon reasonable request.

## Abbreviations

The following abbreviations are used in this manuscript:

VO_2_	Oxygen uptake
VO_2max_	Maximal oxygen uptake
vVO_2max_	Speed corresponding to the VO_2max_ in the incremental test
VCO_2_	Carbon dioxide
H+	Hydrogen ion
Pi	Inorganic phosphate
[La-]	Blood lactate concentration
GET	Gas exchange threshold
vGET	Speed corresponding to the GET in incremental test
50%Δ	Middle point of the difference GET and VO_2max_
v50%Δ	Speed corresponding to 50%Δ in the incremental test
LT	Lactate threshold
VT_1_	First ventilatory threshold
MLSS	Maximal lactate steady state
V_E_	Pulmonary ventilation
RCP	Respiratory compensation point
vRCP	Speed corresponding to the RCP in the incremental test
VT_2_	Second ventilatory threshold
LTP	Lactate turn point
CS	Critical speed
t_Lim_	Time limit
v-t_Lim_	Time-limited model for the assessment of CS using trials with different velocities
d-t_Lim_	Distance-limited model for the assessment of CS using trials with different distances

## References

[1] Burnley M., Jones A.M. (2018). Power–duration relationship: Physiology, fatigue, and the limits of human performance. Eur. J. Sport Sci..

[2] Jones A.M., Burnley M., Black M.I., Poole D.C., Vanhatalo A. (2019). The maximal metabolic steady state: Redefining the ‘gold standard’. Physiol. Rep..

[3] Jones A.M., Wilkerson D.P., DiMenna F., Fulford J., Poole D.C. (2008). Muscle metabolic responses to exercise above and below the “critical power” assessed using ^31^P-MRS. Am. J. Physiol. Regul. Integr. Comp. Physiol..

[4] Sietsema K.E., Rossiter H.B. (2023). Exercise physiology and cardiopulmonary exercise testing. Semin. Respir. Crit. Care Med..

[5] Whipp B.J. (2007). Physiological mechanisms dissociating pulmonary CO_2_ and O_2_ exchange dynamics during exercise in humans. Exp. Physiol..

[6] Broxterman R.M., Ade C.J., Craig J.C., Wilcox S.L., Schlup S.J., Barstow T.J. (2015). The relationship between critical speed and the respiratory compensation point: Coincidence or equivalence. Eur. J. Sport Sci..

[7] Jones A.M., Kirby B.S., Clark I.E., Rice H.M., Fulkerson E., Wylie L.J., Wilkerson D.P., Vanhatalo A., Wilkins B.W. (2021). Physiological demands of running at 2-hour marathon race pace. J. Appl. Physiol..

[8] Poole D.C., Burnley M., Vanhatalo A., Rossiter H.B., Jones A.M. (2016). Critical power: An important fatigue threshold in exercise physiology. Med. Sci. Sports Exerc..

[9] Vanhatalo A., Fulford J., DiMenna F.J., Jones A.M. (2010). Influence of hyperoxia on muscle metabolic responses and the power-duration relationship during severe-intensity exercise in humans: A ^31^P magnetic resonance spectroscopy study. Exp. Physiol..

[10] Zoladz J.A., Majerczak J., Grassi B., Szkutnik Z., Korostyński M., Gołda S., Grandys M., Jarmuszkiewicz W., Kilarski W., Karasinski J. (2016). Mechanisms of attenuation of pulmonary VO_2_ slow component in humans after prolonged endurance training. PLoS ONE.

[11] Massini D.A., Pessôa Filho D.M., Caritá R.A.C., Denadai B.S. (2016). Physiological and perceptual response at critical speed and respiratory compensation point. Braz. J. Sports Med..

[12] Souza K.M., Lucas R.D., Salvador P.C.N., Helal L.C.A.S., Guglielmo L.G.A., Greco C.C., Denadai B.S. (2016). Agreement analysis between critical power and intensity corresponding to 50% in cycling exercise. Braz. J. Kinanthropom Hum. Perform..

[13] Sitko S., Ciper-Sastre R., Corbi F., López-Laval I. (2022). Relationship between functional threshold power, ventilatory threshold and respiratory compensation point in road cycling. J. Sports Med. Phys. Fit..

[14] Pringle J.S.M., Doust J.H., Carter H., Tolfrey K., Campbell I.T., Jones A.M. (2003). Oxygen uptake kinetics during moderate, heavy and severe intensity “submaximal” exercise in humans: The influence of muscle fibre type and capillarisation. Eur. J. Appl. Physiol..

[15] Caputo F., Denadai B.S. (2009). Does 75% of the difference between VO_2_ at lactate threshold and VO_2_max lie at the severe-intensity domain in well-trained cyclists?. Sci. Sports.

[16] Caritá R.A.C., Caputo F., Greco C.C., Denadai B.S. (2013). Aerobic fitness and amplitude of the exercise intensity domains during cycling. Braz. J. Sports Med..

[17] Denadai B.S., Greco C.C. (2017). Can the critical power model explain the increased peak speed/power during incremental test after concurrent strength and endurance training?. J. Strength Cond. Res..

[18] Rossiter H.B. (2011). Exercise: Kinetic considerations for gas exchange. Compr. Physiol..

[19] Lansley K.E., DiMenna F.J., Bailey S.J., Jones A.M. (2011). A ‘new’ method to normalise exercise intensity. Int. J. Sports Med..

[20] Carter H., Pringle J.S., Jones A.M., Doust J.H. (2002). Oxygen uptake kinetics during treadmill running across exercise intensity domains. Eur. J. Appl. Physiol..

[21] Burnley M., Davison G., Baker J.R. (2011). Effects of Priming exercise on VO_2_ Kinetics and the power-duration relationship. Med. Sci. Sports Exerc..

[22] Dekerle J., Baron B., Dupont L., Vanvelcenaher J., Pelayo P. (2003). Maximal lactate steady state, respiratory compensation threshold and critical power. Eur. J. Appl. Physiol..

[23] Pessôa Filho D.M., Alves F., Reis J., Greco C., Denadai B.S. (2012). VO_2_ kinetics during heavy and severe exercise in swimming. Int. J. Sports Med..

[24] Almeida T.A.F., Espada M.C., Massini D.A., Macedo A.G., Castro E.A., Ferreira C.C., Reis J.F., Pessôa Filho D.M. (2023). Stroke and physiological relationships during the incremental front crawl test: Outcomes for planning and pacing aerobic training. Front. Physiol..

[25] Massini D.A., Espada M.C., Macedo A.G., Santos F.J., Castro E.A., Ferreira C.C., Robalo R.A.M., Dias A.A.P., Almeida T.A.F., Pessôa Filho D.M. (2023). Oxygen uptake kinetics and time limit at maximal aerobic workload in tethered swimming. Metabolites.

[26] Broxterman R.M., Ade C.J., Barker T., Barstow T.J. (2015). Influence of pedal cadence on the respiratory compensation point and its relation to critical power. Respir. Physiol. Neurobiol..

[27] Harriss D.J., Jones C., MacSween A. (2022). Ethical standards in sport and exercise science research: 2022 update. Int. J. Sports Med..

[28] Vucetić V., Sentija D., Sporis G., Trajković N., Milanović Z. (2014). Comparison of ventilation threshold and heart rate deflection point in fast and standard treadmill test protocols. Acta Clin. Croat.

[29] Scheuermann B.W., Kowalchuk J.M. (1998). Attenuated respiratory compensation during rapidly incremented ramp exercise. Respir. Physiol..

[30] Massini D.A., Caritá R.A.C., Siqueira L.O., Simionato A.R., Denadai B.S., Pessôa Filho D.M. (2018). Assessment of critical speed in track and treadmill: Physiological profiles and relationship with 3000-meter performance. Braz. J. Kinanthropom Hum. Perform..

[31] Díaz S.P., Fernández S.P. (2002). Determinación del tamaño muestral para calcular la significación del coeficiente de correlación lineal. Cad. Aten. Primaria.

[32] Bland J.M., Altman D.G. (1986). Statistical methods for assessing agreement between two methods of clinical measurement. Lancet.

[33] Ferguson C.J. (2009). An effect size primer: A guide for clinicians and researchers. Prof. Psychol. Res..

[34] Vanhatalo A., Black M.I., DiMenna F.J., Blackwell J.R., Schmidt J.F., Thompson C., Wylie L.J., Mohr M., Bangsbo J., Krustrup P. (2016). The mechanistic bases of the power-time relationship: Muscle metabolic responses and relationships to muscle fibre type. J. Physiol..

[35] Murgatroyd S.R., Wylde L.A., Cannon D.T., Ward S.A., Rossiter H.B. (2014). A ‘ramp-sprint’ protocol to characterise indices of aerobic function and exercise intensity domains in a single laboratory test. Eur. J. Appl. Physiol..

[36] Philp A., Macdonald A., Carter H., Watt P., Pringle J. (2008). Maximal lactate steady state as a training stimulus. Int. J. Sports Med..

[37] Neder J.A., Jones P.W., Nery L.E., Whipp B.J. (2000). The effect of age on the power/duration relationship and the intensity-domain limits in sedentary men. Eur. J. Appl. Physiol..

[38] Busso T., Chatagnon M. (2006). Modelling of aerobic and anaerobic energy production in middle-distance running. Eur. J. Appl. Physiol..

